# Ketogenic Ratio Determines Metabolic Effects of Macronutrients and Prevents Interpretive Bias

**DOI:** 10.3389/fnut.2018.00075

**Published:** 2018-08-30

**Authors:** Tanya Zilberter, Yuri Zilberter

**Affiliations:** ^1^Infotonic Consultancy, Marseille, France; ^2^Inserm UMR 1106, Marseille, France

**Keywords:** diet classification, ketogenic threshold, anti-ketogenic threshold, macronutrients, metabolic effects of diets

## Introduction

Thomas Seyfried remarked in his book [(1), page 6]: “The definition of ketogenic diet allows for considerable leeway in food choices as long as the individual has reduced blood glucose and is producing ketones.” Unfortunately, these parameters are lacking in many if not most of studies into metabolic effects of macronutrients. Meanwhile, there is a precise way to predict whether or not a diet will induce ketosis and the aim of this opinion article is to advocate a broader usage of this way. Why is this so important?

Excess of carbohydrate intake typical for consumers of the Western diet may cause detrimental effects on metabolism and increase risks of the onset and progression of many neurodegenerative diseases (2–4). On the other hand, diets high in fat and low in carbohydrates decrease appetite, probabilities of food addiction and obesity, and are neuroprotective (5, 6). Carbohydrate restriction induces physiological changes which are very similar to the well documented beneficial effects of calorie restriction (7, 8). Conversely, the hallmark of high-carbohydrate diets is homeostatic inadequacy (9), an overproduction of reactive oxygen species and advanced glycation products, both of which are implicated in neuroinflammation and neurodegeneration (10–12). However, the meaning of “high” or “low” in diets' definition has been drifting away from the previously established quantitative criterion known as ketogenic ratio.

## The ketogenic ratio

Almost a century ago, Woodyatt (13) wrote: “antiketogenesis is an effect due to certain products which occur in the oxidation of glucose, an interaction between these products on the one hand and one or more of the acetone bodies on the other.” The ketogenic ratio (KR), as proposed by Shaffer (14), is a ratio of the sum of ketogenic factors to the sum of antiketogenic factors: KR = K/AK. The antiketogenic part of the equation invariably equals 1 so the KRs are always expressed as 2:1, 4:1, etc. For the sake of economy of reading, we leaved out the repeating part not bearing any information and mention only the informative digit.

Shaffer concluded that the maximal ratio compatible with the oxidation of the “ketogenic” molecules becomes possible at the KR = 1, making KRs below 1 antiketogenic and KRs above 2 ketogenic. Wilder and Winter (15) described the KR of a food in terms of times the fat content exceeds the amount of carbohydrate and protein combined, roughly. The reasoning was based on their own experimental observation that fats are predominantly ketogenic (90%), carbohydrates are almost 100% anti-ketogenic, and protein is both ketogenic and antiketogenic, 46–58% respectively. They arrived, along with Woodyatt and Sansum (13), at the conclusion that KR for induction of ketogenesis should be 2 or higher while the upper limit of antiketogenesis is 1.

In 1980, Withrow (16) modified the equation and since that time, the equation looked like this:

KR = (0.9 F + 0.46 P): (C + 0.58 P + 0.1 F) where F is grams of fat; P is grams of protein and C is grams of carbohydrate. Currently, this equation is rarely used in nutrition research and less so in dietetic practice, which is regrettable since properly calculated KRs reveals interesting patterns of diet effects. Previously (9), using the Withrow's equation, we calculated KRs in a number of diets and came to conclusion that the watershed in the group of effects occurs at KR of about 1.7. Above this value, metabolic features of diets were characteristic for ketogenesis, while below this value, they were characteristic for the obesogenic high-fat diet (oHFD), which, in contrast to the diet resulting in ketosis (KD), is high in fat but also in carbohydrates. Here, we analyzed three groups of diets in order to compare our observation regarding the watershed with the classification of diets made by the authors of 62 studies, in which it was possible to calculate KRs.

## Uncertainty in current diet labelling

**Figure 1:**
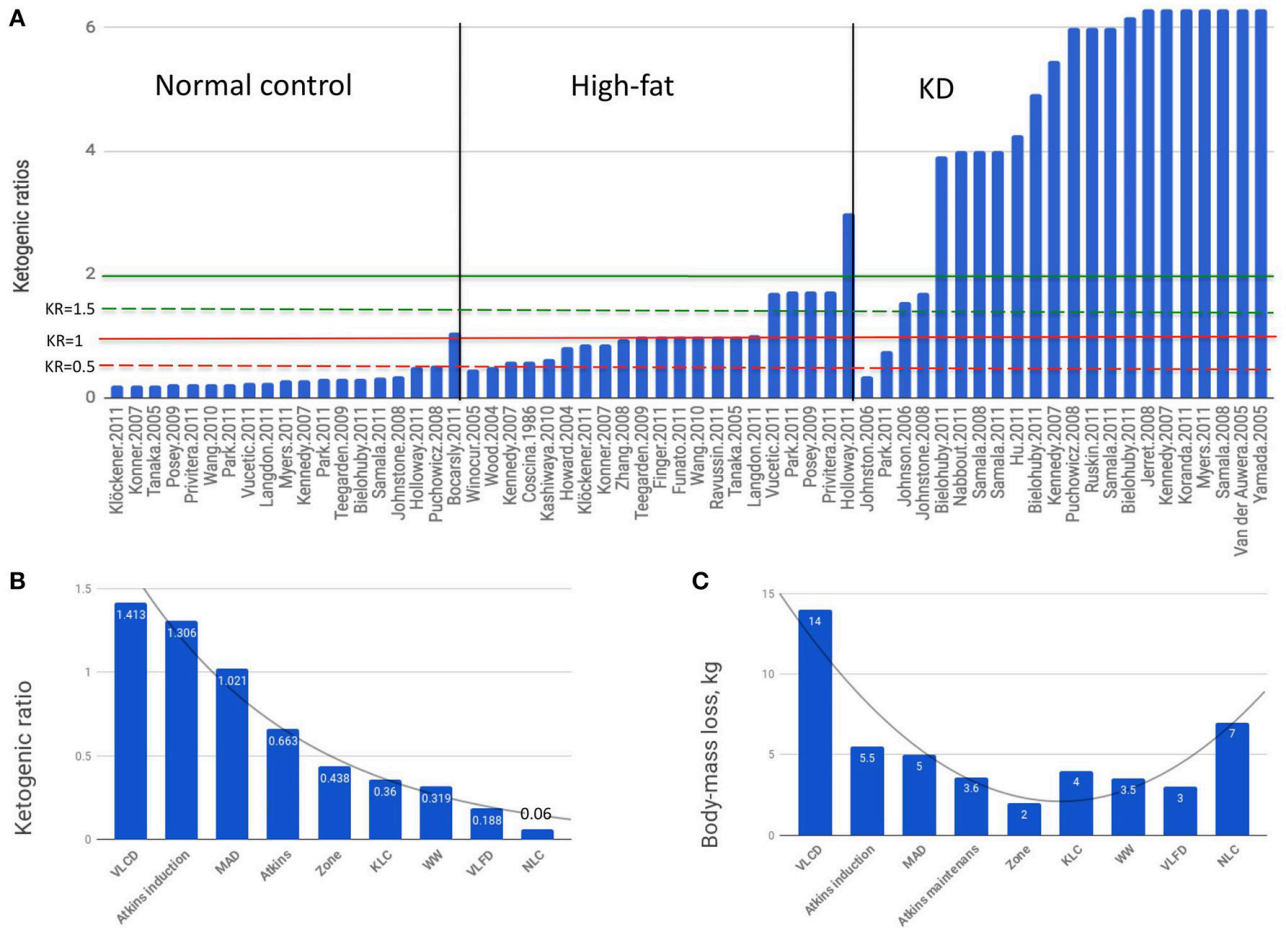
**(A)** Green line, theoretical threshold of ketogenesis; dashed green line, empirical threshold of ketogenesis. Red line, theoretical threshold of anti-ketogenesis; dashed red line KR = 0.5. Vertical axis, ketogenic ratios. Horizontal axix, original studies, first author and year (17–50). **(B)** Ketogenic ratios of diets below the ketogenic threshold. Calculated basing on data extracting from the studies (25, 51–54). Gray curves in **(B,C)** trendlines. **(C)** Body-mass loss on diets below the ketogenic threshold. VLCD, very low carbohydrate diet; MAD, modified Atkins diet; Zone, the Zone diet; KLC, ketogenic low carbohydrate diet; WW, Weight Watchers diet; VLFD, very low fat diet; NLC, non-ketogenic low carbohydrate diet.

We can see that there is no common criteria in choosing diet compositions for the “normal control” group to start with (Figure 1A). The vast majority of “normal control” diets are clearly anti-ketogenic (KRs below or equal 1). The oHFD group of diets had broader spectrum of KRs ranging from the anti-ketogenic 0.456 to clearly ketogenic 2.994. The KRs of diets, which were considered ketogenic by the authors, ranged especially broadly: from 0.36 to above 6. The macronutrient compositions of oHFD diets and KDs overlap although the obesogenic oHFD is discussed in literature as diametrically different from the KD. The metabo- and neuroprotective effects of KD are experimentally and clinically confirmed, however, the low compliance rate of the strict KD caused mass attempts to reduce the KR below KR = 2, which for a century used to be the minimal accepted value to consider a diet ketogenic.

An example is the Modified Atkins Diet (MAD) first tried at The Johns Hopkins Hospital. It is a protocol replicating the induction phase of the original Atkins diet. MAD is composed of approximately 10% energy from carbohydrates, 30% from protein, and 60% from fat, no calorie restriction (55–58). The MAD became an intervention for treating a number of diseases, first of all in cases of intractable childhood epilepsy but also in pharmacoresistant epilepsy in adults and in pathologies of glucose utilization (58). MAD is labeled “ketogenic” while its KR = 1.3 (calculated basing on 11). However, its efficiency is limited: although 70% of epilepsy patients on MAD experienced a 50% reduction in seizures, after switching to the strict clinical KD (KR = 4) the patients benefited from an additional 37% improvement and 18.5% became seizure-free (13, 59, 60) indicating that the KR is indeed an important predictor of diet effectiveness.

## Effects of KD vs. oHFD

Basing on our review of KR-dependent effects of diets (6), here we compiled a brief overview in order to demonstrate the critical differences between KD and oHFD.

1. The risks of pathologies caused by brain hypometabolism (e.g., due to hypoxia, hypoglycemia, brain trauma) is reduced in diets capable of inducing ketogenesis; the opposite is shown for the oHFD diets (39, 61–69).
2. Inflammation, e.g., neuroinflammation is shown to be induced by oHFD but decreased by KD (70–78) among other things resulting in improved or impaired cognitive function (76, 78–82).
3. Neuronal hyperactivity and epilepsy is attenuated by KD but exacerbated by oHFD (75, 83–85, 85–88).
4. Inhibition of growth of tumor and metastasis as well as tumor neo-angiogenesis is demonstrated for KD while oHFD increases the risk of cancerogenesis (8, 89–92).
5. KD decreased cardiovascular risks while oHFD increases them (93, 94).
6. KD lowered the type 2 diabetes risks, improves management of complications and glucose control while oHFD increases the risks, exacerbates complications and induced glucose intolerance (95–99).

## Below the ketogenic threshold

In the ragne of KRs between 1 and 2 lays the area of metabolic uncertainty where the effects are poorly predictable, the definitions are vague and outcomes even more so. The most critical value in this area is 1.5, the experimentally reached threshold of anti-ketogenesis (100). Here we report the result of our analysis of non-ketogenic diets (Figures 1B,C) using data extracted from the studies:

- Very low carbohydrate diet [VLCD, (51)];
- The Atkins diet - induction and maintaining phases, the Zone diet and very low fat diet, [VLFD, (52, 53)];
- MAD, Zone, Weight Watchers diet (WW) and VLFD (54);
- Ketogenic low carbohydrate diet (KLC) and non-ketogenic low carbohydrate diet (NLC) (25).

The common feature of these diets was that none of them reached the threshold of ketogenesis defined as KR = 1.5 (100). The diets were roughly isocaloric (1,412 ± 35.5 Kcal/day; caloric intake of WW varied averaging 1,400 Kcal), with main outcome a body-mass loss. Beyond the strictly utilitarian standpoint, body-mass loss is an indicator of lipolysis and thus of ketogenesis likelihood.

In diets ranging from KR = 1.413 to KR = 0.06 (Figure 1B), the metabolic outcome did not depend on KR as directly as it does above the ketogenic threshold (Figure 1C) indicating that mechanisms other than the ketogenesis-glycolysis counterbalancing seem to be predominant. Indeed, in the study of Johnston et al. (25) the inverse relationship has been observed: the KLC diet having six times higher KR than NLC (0.35 vs. 0.06) had almost twice lower effect (4 vs. 7 kg) leading the authors to conclusion they even used as the article title: “*Ketogenic low-carbohydrate diets have no metabolic advantage over nonketogenic low-carbohydrate diets*”—although both diets were undoubtedly anti-ketogenic.

It has been shown before (26) that the prevalence of carbohydrates in an otherwise equally high-protein diets increased energy intake in the *ad libitum* consumption mode initiating the vicious cycle of non-homeostatic processes, including reward seeking and food addiction (101) and the prevalence of energy-conserving metabolic mode over the homeostatically balanced mode (9). Theoretically, below the KR = 1.5, glucocentric metabolic mode prevails while above KR = 1.5 the dominant metabolic mode is adipocentric with the important consequence being initiation of lipolysis (102). This is why the body-mass loss of diets is a convenient parameter indirectly indicating that lipolytic processes take place. It has been measured empirically (100) that ketosis is not observed in KRs below 1.5—however, the “ketogenic” label has been assigned to MAD (KR = 1.021) and even to KLC (KR = 0.36).

There is a turning point in the KR-effect interaction curve at KR = 0.5 (Figure 1C), the diet nearest to this point being at KR = 0.438 [the Zone diet, (52–54)]. Further decrease of KRs up to the value of 0.06 resulted in an inverted U-shaped dose-response relationship under the threshold of ketogenesis.

## Discussion

Currently the classification of diets is rather unsatisfactory and diet labels offered by the authors (quite arbitrarily) “normal”, “oHFD,” or “ketogenic” oftentimes do not correspond to their respective diets' macronutrient compositions. Description based on percentages of energy from each of the macronutrients does not make it easy to qualify diet type and unify the categorization. The macronutrient ratio in terms of ketogenicity is often ignored in qualification of metabolic effects. The most striking example of this is the oHFD diet, which in fact is also high in carbohydrates. As we briefly discussed above, its effects are diametrically opposing those of the ketogenic diet which is also high in fat but low in carbohydrates, resulting in striking differences in diets' physiological effects (see *Effects of KD* vs. *oHFD*).

The a*d libitum* access to food is the standard protocol in animal experiments although the validity of it is rightfully questioned since the subjects become “*sedentary, obese, glucose intolerant, and on a trajectory to premature death*” [(103), page 6,127]. On the other hand, the low-carbohydrate diets that are high in fat have a number of metabolic advantages: for instance, they facilitate increase energy expenditure by increasing thermogenic effects and excretion of ketone bodies (104).

Greater carbohydrate intake was associated with poorer performance in patients with Alzheimer's disease (105), while KD improved cognition independent of weight loss in healthy human subjects (80). KD improved verbal vocabulary and reaction time in children with epilepsy and attention (4), concentration, and memory in adults with multiple sclerosis (106). Diets limiting carbohydrate intake mimic the effects of fasting or caloric restriction (102). In fact, calorie restriction is not even required on a very low carbohydrate diet to achieve the desired goals, while on a low-fat, high-carbohydrate diet calorie restriction is the principal requirement (107).

The metabolic effects of dietary fat on energy homeostasis differs from the effects of carbohydrates in two key features. One is the ability to store energy in depots - fat is exceptionally good at it, but carbohydrates are limited in this ability. The other is the ability to increase the drive to consume energy. Carbohydrates have a characteristic ability to elicit positive reward and thus addiction (5, 101, 108–110) while significant carbohydrate restriction in VLCD caused not only energy intake decrease but also energy expenditure increase in both resting and active states (51). In spite of these non-homeostatic features, these mechanisms are evolutionarily appropriate in wild nature, but as soon as the living conditions change the hard-wired pursuit to maximize the energy store becomes a metabolic trap (9s), resulting in non-homeostatic overconsumption and all the negative metabolic consequences it causes.

To conclude, the current classification of diets results in terminological confusion. We suggest that rethinking the existing descriptive approach and reanimating the century-old qualitative and clear-cut criterion may facilitate the use of common language and substantive discussion in nutrition and metabolism.

## Author contributions

TZ and YZ equally contributed to the concept, data collection and analysis, writing the manuscript and preparing illustrations.

### Conflict of interest statement

The authors declare that the research was conducted in the absence of any commercial or financial relationships that could be construed as a potential conflict of interest.
